# Supplementary material for: Transcriptomic and metabolomic analysis of copper stress acclimation in Ectocarpus siliculosus highlights signaling and tolerance mechanisms in brown algae
Source: BMC Plant Biol. 2014 May 1;14:116. doi: 10.1186/1471-2229-14-116 (PMC4108028; doi:10.1186/1471-2229-14-116)
Supplement: Additional file 2 — Venn diagram representing the number of significantly up-regulated (a) and down-regulated (b) contigs/singletons under copper (Cu), oxidative, hypersaline (Hyper), and hyposaline (Hypo) stress conditions (p < 0.05). [file 1471-2229-14-116-S2.pdf]

Additional file 2

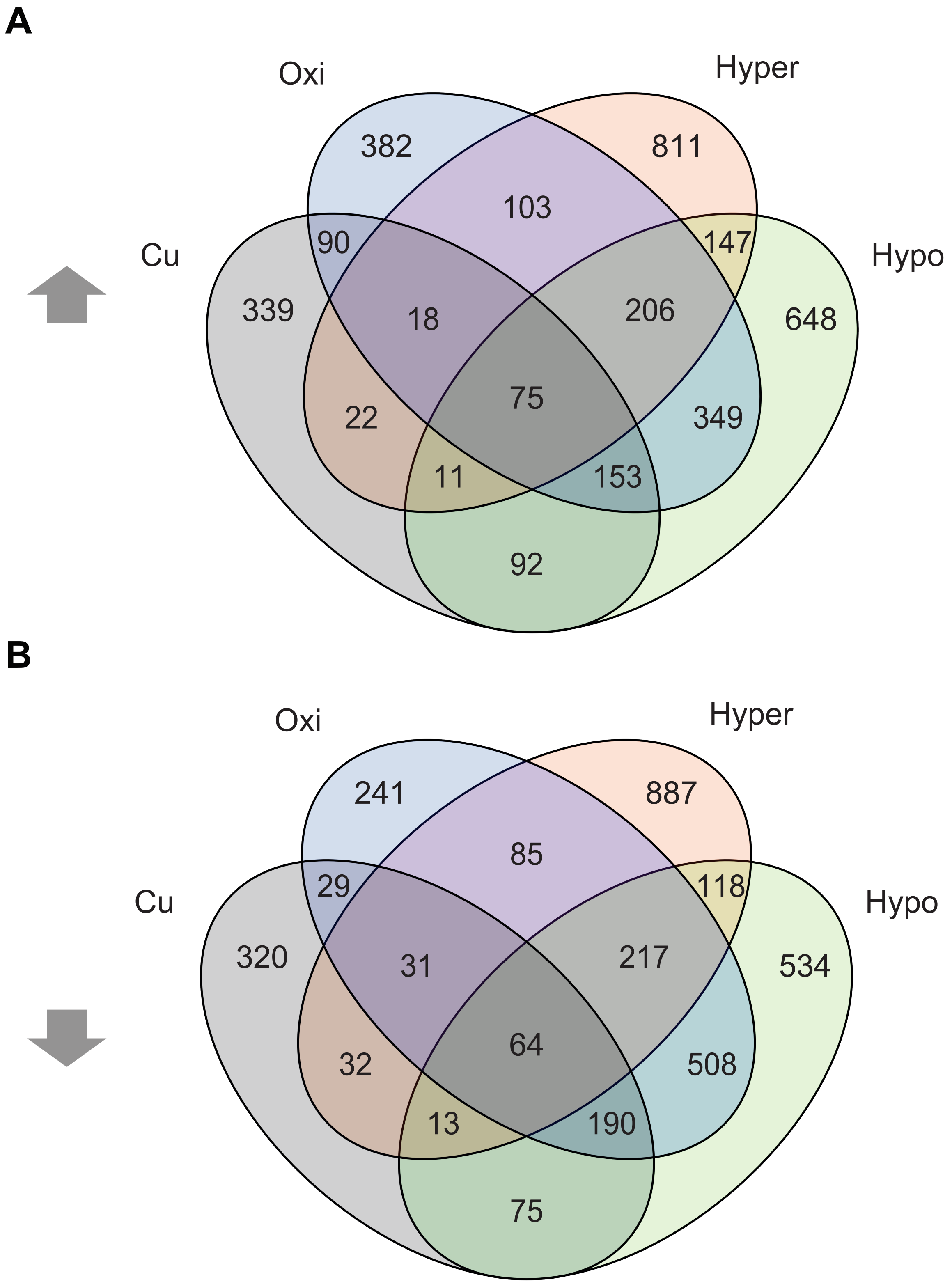

Additional file 2. Venn diagram representing the number of up-regulated (a) and down-regulated (b) contigs/singletons under copper (Cu), oxidative (Oxi), hypersaline (Hyper), and hyposaline (Hypo) stress conditions ($p < 0.05$).
