# Supplementary material for: Transcriptomic and metabolomic analysis of copper stress acclimation in Ectocarpus siliculosus highlights signaling and tolerance mechanisms in brown algae
Source: BMC Plant Biol. 2014 May 1;14:116. doi: 10.1186/1471-2229-14-116 (PMC4108028; doi:10.1186/1471-2229-14-116)
Supplement: Additional file 4 — Hierarchical clustering of gene expression data obtained by microarray in E. siliculosus submitted to different abiotic stress conditions. Hypo_1-4 = hyposaline stress (salinity of 4 ppt, 6 h), Hyper_1-4 = hypersaline stress (salinity of 96 ppt, 6 h), Oxi_1-4 = oxidative stress (10 mM H2O2, 6 h; see Dittami et al.[25] for experimental details for these stressors), and Cu_1_4h - Cu_3_8h (250 μg L-1 copper stress, 4–8 h; this study). The heat map shows log2-ratios of gene expression in stress compared to control conditions. Gene trees and sample trees were obtained by hierarchical clustering and “Euclidean distance” as metric. [file 1471-2229-14-116-S4.pdf]

Additional file 4

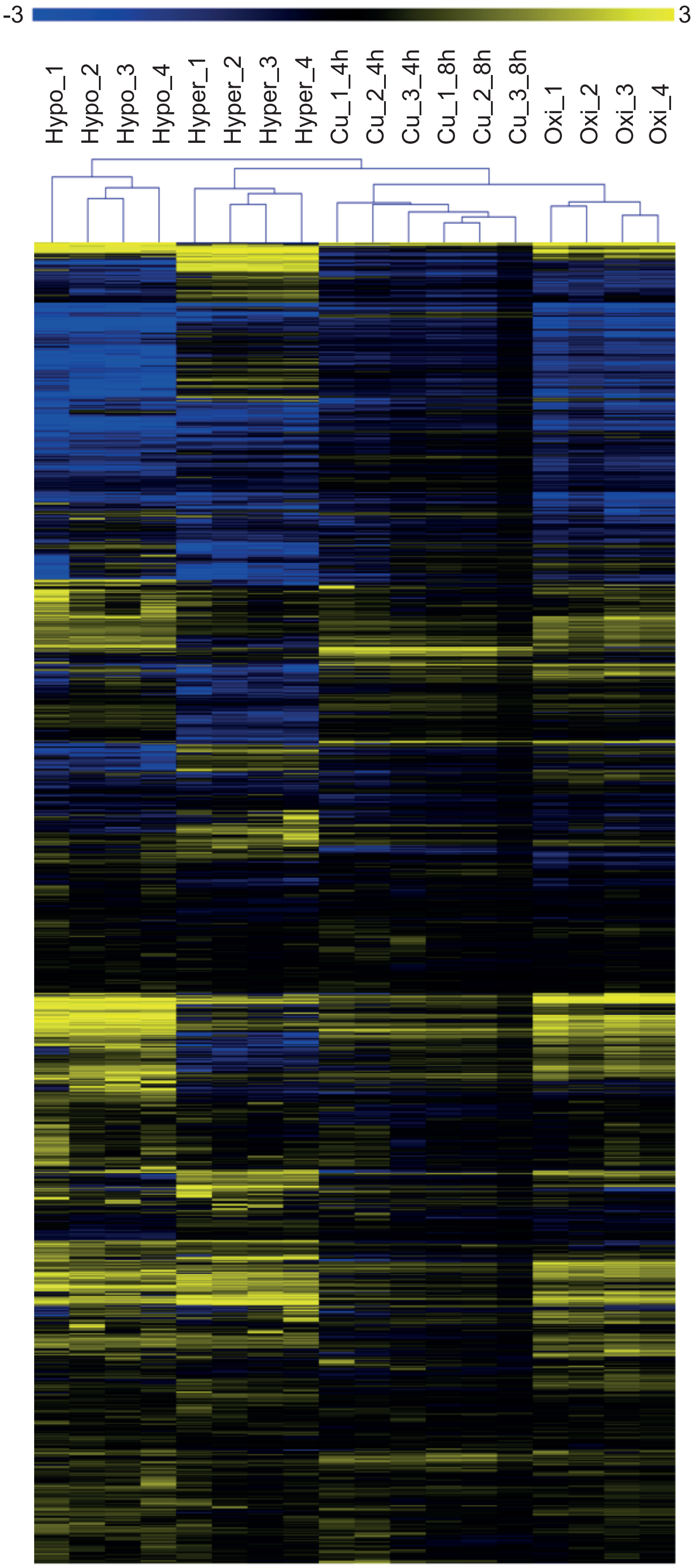

Additional file 4. Hierarchical clustering of gene expression data obtained by microarray in *E. siliculosus* submitted to different abiotic stress conditions: Hypo_1-4 = hyposaline stress (salinity of 4 ppt, 6 h), Hyper_1-4=hypersaline stress (salinity of 96 ppt, 6 h), Oxi_1-4=oxidative stress (10mM H_2O_2, 6 h, see Dittami *et al.* 2009 for experimental details for these stressors), and Cu_1_4h - Cu_3_8h (250 µg L^{-1} copper stress, 4-8 h, this study). The heat map shows log2-ratios of gene expression in stress compared to control conditions. Gene trees and sample trees were obtained by hierarchical clustering and “Euclidean distance” as metric.
