# Supplementary material for: Transcriptomic and metabolomic analysis of copper stress acclimation in Ectocarpus siliculosus highlights signaling and tolerance mechanisms in brown algae
Source: BMC Plant Biol. 2014 May 1;14:116. doi: 10.1186/1471-2229-14-116 (PMC4108028; doi:10.1186/1471-2229-14-116)
Supplement: Additional file 5 — (A) ClustalW multiple alignment of the CYP74 family IHCD domains from Esi0060_0078 and homologs. The conserved catalytic residues FXXXFXSXX were highlighted in red for the CYP74A conserved phenylalanine residues and blue for the AOS specific serine residue. (B) Ribbon diagram of a predicted structural model of Esi0060_0078 prepared with the Phyre2 software [73]. [file 1471-2229-14-116-S5.pdf]

Additional file 5

A

Enzyme subfamily	Genbank Id	Protein region	Clustal W of the IHCD motif
Esi0060_0078	CBN7495	226	FMFTTNFQSAGAIA
ZmAOS (CYP74A)	AAR33048	272	LLFATTFNSYGGLK
LeAOS (CYP74A)	AAF67141	308	FVFLAGFNSYGGLK
OsAOS (CYP74A)	AAP50956	308	ILFALCFNSYGGFK
LuAOS (CYP74A)	AAA03353	332	ILFAVCFNSWGGFK
MtAOS (CYP74A)	CAC86897	320	LLFATCFNSFGGMK
PaAOS (CYP74A)	CAA55025	269	ILFAVCFNTFGGFK
PgHPL (CYP74B)	AAK15070	298	LLFILGFNAFGGFS
LeHPL (CYP74B)	AAF67142	280	LLFILGFNAFGGFS
OsHPL (CYP74C)	EAZ22254	281	LVFVAIFNAFGGFK
MtHPL (CYP74C)	CAC86899	282	IIFTAGFNAYGGLK
NtDES (CYP74D)	AAL40900	276	ILFLVGINMFAGLN
CaDES (CYP74D)	ABH03632	275	MLFLVGINMFAGLN

B

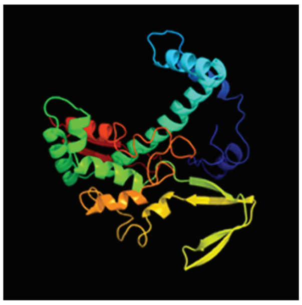

Image coloured by rainbow N → C terminus

**Aligned:**	**260 aa**
**Modeled:**	**269 aa**
**Confidence:**	**100%**
**% Coverage:**	**91%**
**% Identity:**	**18%**
**PDB header:**	**lyase**
**Model based on template:**	**C3danA**
**Chain: A:**	**PDB Molecule: cytochrome p450 74a2**
**Molecule:**	**Cytochrome p450 74a2**
**PDBTitle:**	**Crystal structure of allene oxide synthase**

Additional file 5. (A) ClustalW multiple alignment of the CYP74 family IHCD domains from Esi0060_0078 and homologs. The conserved catalytic residues FXXXFXSXX were highlighted in red for the CYP74A conserved phenylalanine residues and blue for the AOS specific serine residue. (B) Ribbon diagram of a predicted structural model of Esi0060_0078 prepared with the Phyre2 software [73].
