# Supplementary material for: Transcriptomic and metabolomic analysis of copper stress acclimation in Ectocarpus siliculosus highlights signaling and tolerance mechanisms in brown algae
Source: BMC Plant Biol. 2014 May 1;14:116. doi: 10.1186/1471-2229-14-116 (PMC4108028; doi:10.1186/1471-2229-14-116)
Supplement: Additional file 6 — Summary of features observed in the putative E. siliculosus Cu induced heavy metal P 1B -ATPase (Esi0023_0054). This sequence contains conserved heavy metal-associated (HMA), E1-E2 ATPase superfamily and haloacid dehalogenase (HAD) domains. The diagram was obtained by amino acid sequence analysis based on the Conserved Domains and Protein Classification Database. [file 1471-2229-14-116-S6.pdf]

Additional file 6

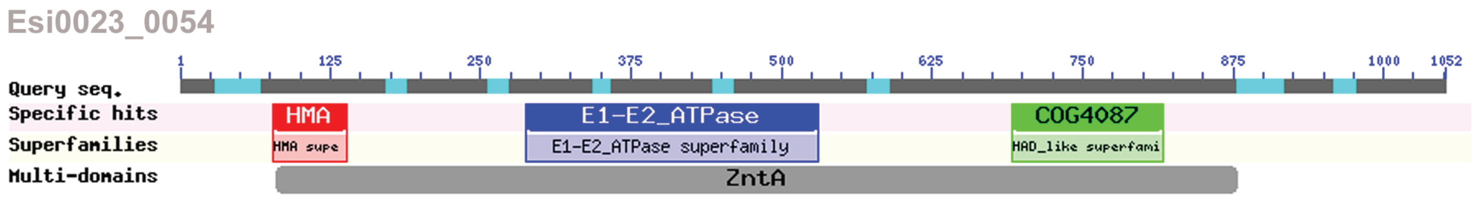

Additional file 6. Summary of features observed in the putative *E. siliculosus* Cu induced heavy metal P1B-ATPase (Esi0023_0054). This sequence contains conserved heavy metal-associated (HMA), E1-E2 ATPase superfamily and haloacid dehalogenase (HAD) domains. The diagram was obtained by amino acid sequence analysis based on the Conserved Domains and Protein Classification Database.
