# Supplementary material for: Transcriptomic and metabolomic analysis of copper stress acclimation in Ectocarpus siliculosus highlights signaling and tolerance mechanisms in brown algae
Source: BMC Plant Biol. 2014 May 1;14:116. doi: 10.1186/1471-2229-14-116 (PMC4108028; doi:10.1186/1471-2229-14-116)
Supplement: Additional file 7 — Results of partial least squares discriminant analysis (PLS-DA) carried out for the compounds detected in the Cu-stress experiment. The plot represents the variations of 392 monoisotopic peaks quantified by UPLC-MS in positive ion mode in algal samples harvested after 4 h and 8 h of Cu-stress (red and yellow spots, respectively), as well as the corresponding controls (green and blue spots). [file 1471-2229-14-116-S7.pdf]

Additional file 7

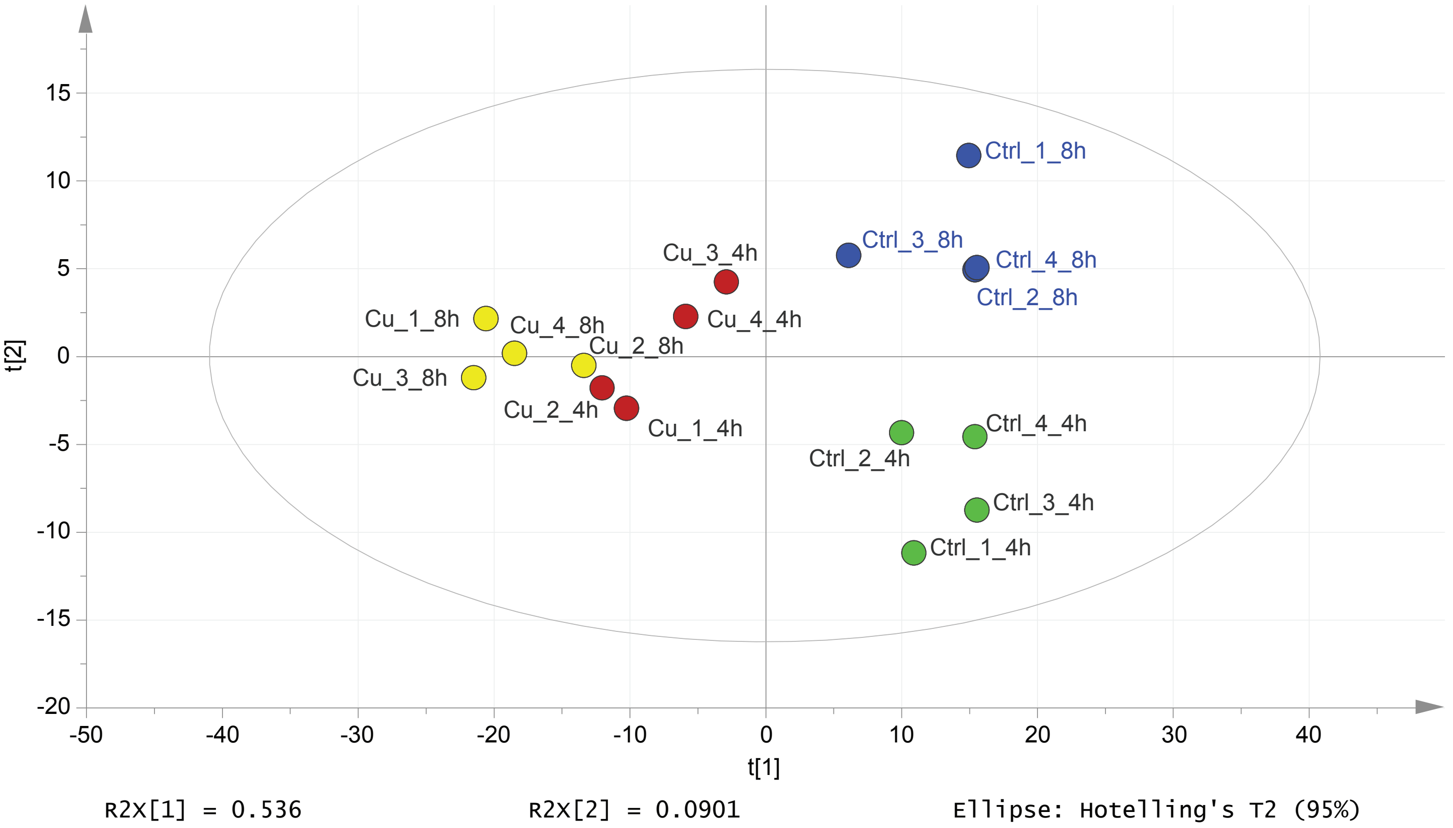

Additional file 7. Results of partial least squares discriminant analysis (PLS-DA) carried out for the compounds detected in the Cu-stress experiment. The plot represents the variations of 392 monoisotopic peaks quantified by UPLC-MS in positive ion mode in algal samples harvested after 4 h and 8 h of Cu-stress (red and yellow spots, respectively), as well as the corresponding controls (green and blue spots).
