# Supplementary material for: Transcriptomic and metabolomic analysis of copper stress acclimation in Ectocarpus siliculosus highlights signaling and tolerance mechanisms in brown algae
Source: BMC Plant Biol. 2014 May 1;14:116. doi: 10.1186/1471-2229-14-116 (PMC4108028; doi:10.1186/1471-2229-14-116)
Supplement: Additional file 8 — Hierarchical clustering of 392 monoisotopic peaks quantified by UPLC-MS in positive and negative ion mode in algal samples under copper stress and control conditions. Concentrations of each metabolite were normalized to a maximum of 1 (see Methods) and clustering was carried out with the Euclidean distance matrix. [file 1471-2229-14-116-S8.pdf]

Additional file 8

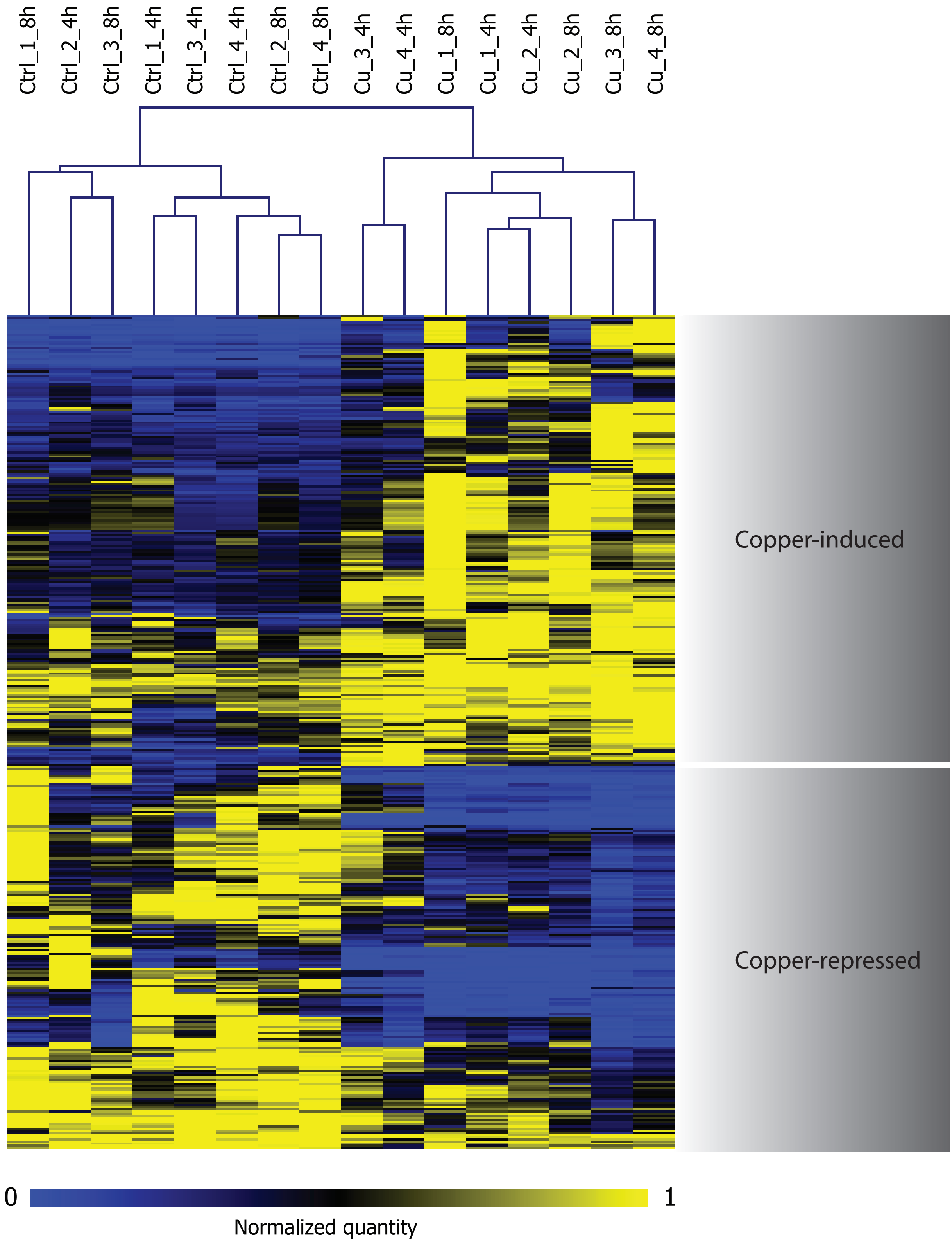

Additional file 8. Hierarchical clustering of 392 monoisotopic peaks quantified by UPLC-MS in positive and negative ion mode in algal samples under copper stress and control conditions. Concentrations of each metabolite were normalized to a maximum of 1 (see Materials and Methods) and clustering was carried out with the Euclidean distance matrix.
