# Supplementary material for: Transcriptomic and metabolomic analysis of copper stress acclimation in Ectocarpus siliculosus highlights signaling and tolerance mechanisms in brown algae
Source: BMC Plant Biol. 2014 May 1;14:116. doi: 10.1186/1471-2229-14-116 (PMC4108028; doi:10.1186/1471-2229-14-116)
Supplement: Additional file 9 — Results of PLS-DA representing the variations of (A) amino acid (GC-MS analysis) and (B) fatty acid contents (UPLC-MS analysis) between control and Cu treated samples. [file 1471-2229-14-116-S9.pdf]

Additional file 9

A

PLS-DA (SIMCA-P) on amino acids

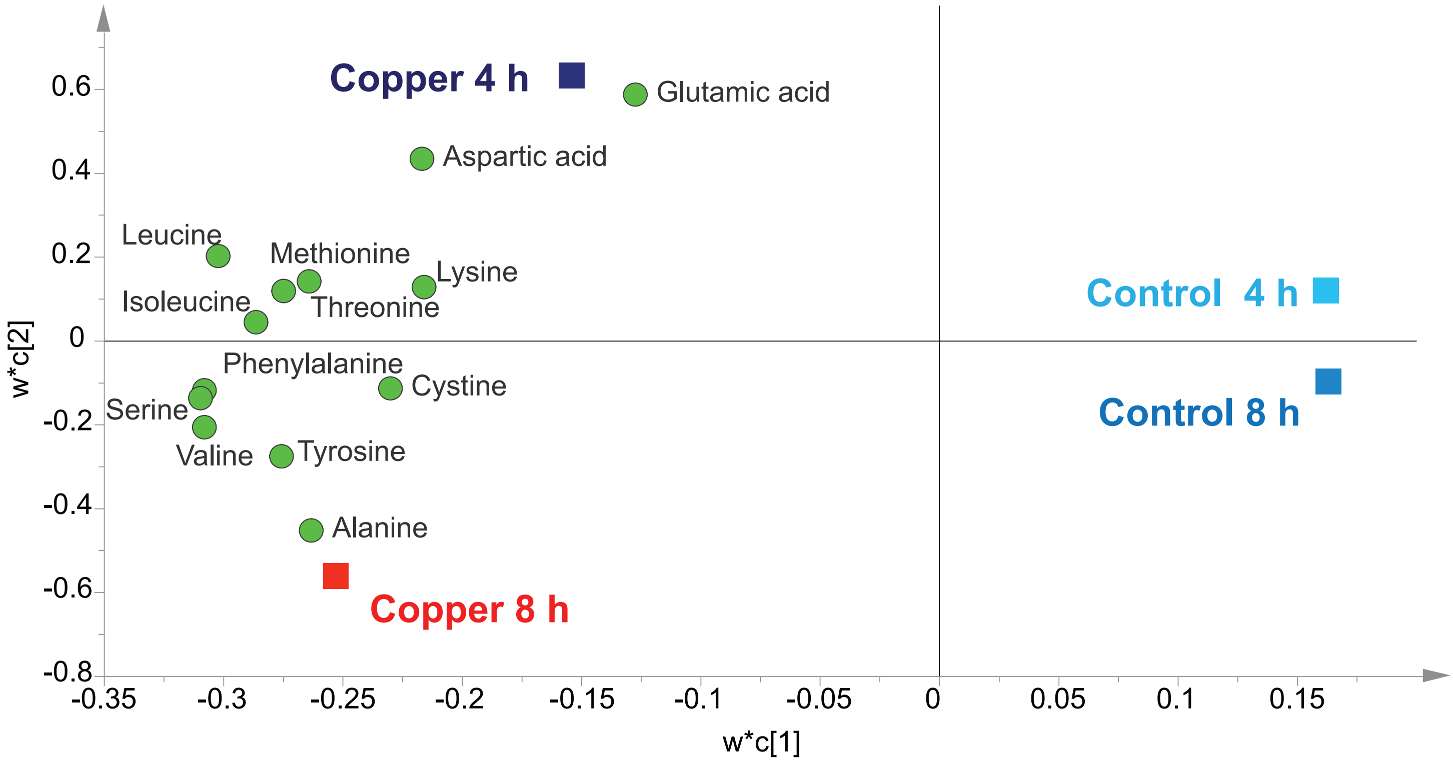

B

PLS-DA (SIMCA-P) on fatty acids

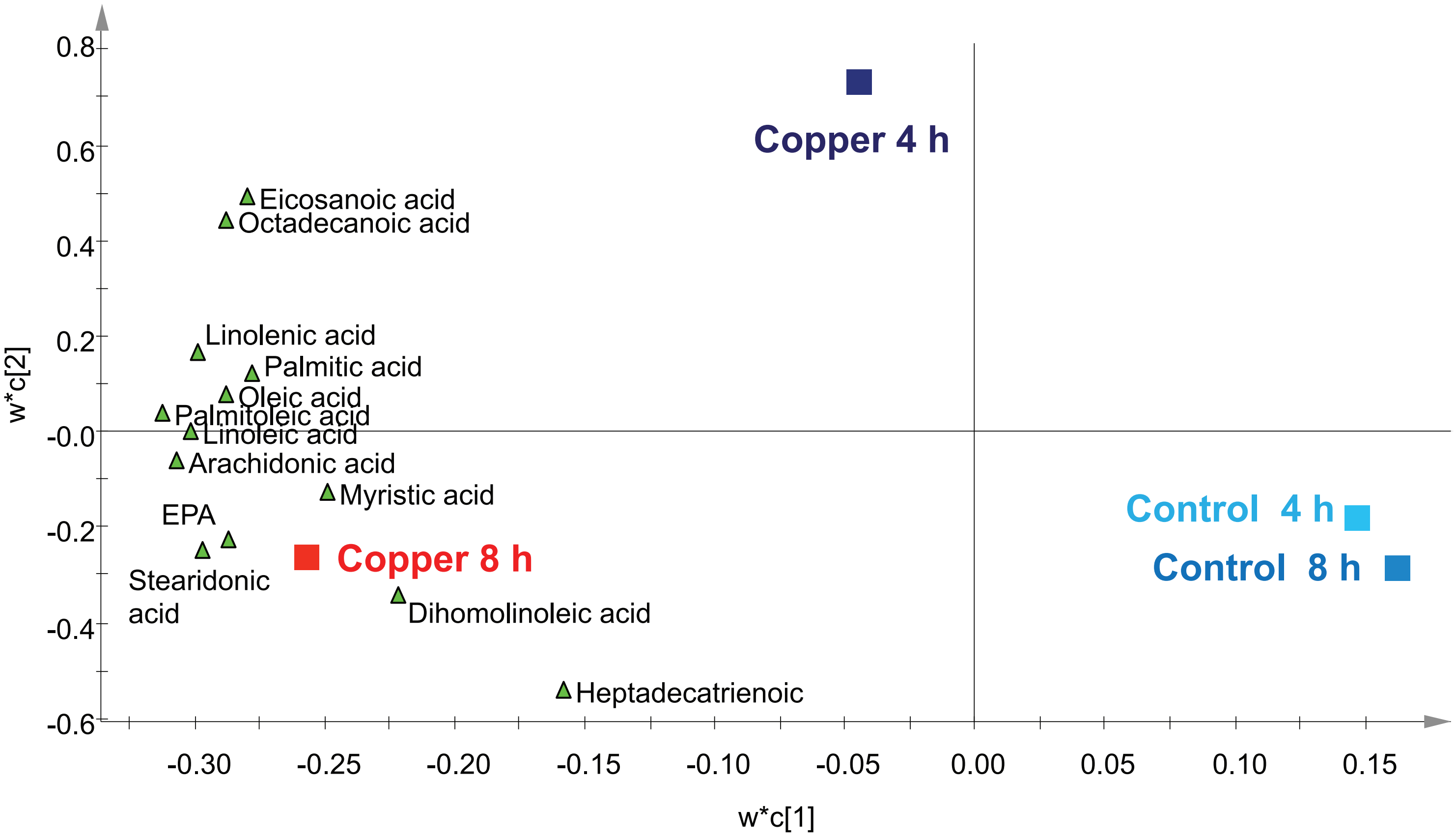

Additional file 9. Results of PLS-DA representing the variations of (A) amino acid (GC-MS analysis) and (B) fatty acid contents (UPLC-MS analysis) between control and Cu treated samples.
