# Supplementary material for: Transcriptomic and metabolomic analysis of copper stress acclimation in Ectocarpus siliculosus highlights signaling and tolerance mechanisms in brown algae
Source: BMC Plant Biol. 2014 May 1;14:116. doi: 10.1186/1471-2229-14-116 (PMC4108028; doi:10.1186/1471-2229-14-116)
Supplement: Additional file 11 — Parameters used for XCMS analysis. [file 1471-2229-14-116-S11.pdf]

Additoinal file 11: Detailed parameters used for XCMS analysis

---

Software:

- XCMSOnline version: 0.0.84
- XCMS version: 1.30.3

1. General parameters

- Polarity: positive
- retention time format: minutes

2. Feature detection

- method: centWave
- ppm: 5
- snthr: 6
- peakwidth: 5 60
- mzdiff: 0.01
- prefilter peaks: 3
- prefilter intensity: 100
- noise: 0

3. Retention time correction

- method: obiwarp
- profStep: 0.1

4. Alignment

- method: density
- bw: 20
- mzwid: 0.015
- minfrac: 0.7
- minsamp: 1

5. FillPeaks

6. Diffreport

- statistics.threshold.pvalue: 0.05
- statistics.diffReport.value: into

7. Additional Plots & Statistics

8. Annotation (isotopes & adducts)

- featureAnnotation.CAMERA.annotate: isotopes
- featureAnnotation.CAMERA.mzabs: 0.015
- featureAnnotation.CAMERA.ppm: 5
- featureAnnotation.CAMERA.sigma: 6
- featureAnnotation.CAMERA.perfwhm: 0.6
- featureAnnotation.CAMERA.maxcharge: 3
- featureAnnotation.CAMERA.maxiso 4
- featureAnnotation.CAMERA.intensity into

9. Putative ID's (METLIN)

- identification.METLIN.ppm: 5
- identification.METLIN.adducts: " M+H, M+Na "
